# Structuring Digital Mental Health Care Navigation: Co-Design Nominal Group Technique Study to Develop the MChart Definition and Typology of the Characteristics of Digital Mental Health Care Navigation Tools

**DOI:** 10.2196/91462

**Published:** 2026-07-27

**Authors:** Jane Koerner, Luis Salvador-Carulla, Cindy Woods, Sebastian Rosenberg, MaryAnne Furst, Sue Lukersmith, Hossein Tabatabaei-Jafari, Amir Aryani, MChart Expert Panel

**Affiliations:** 1 Health Research Institute University of Canberra Bruce, Australian Capital Territory Australia; 2 National Centre for Epidemiology and Population Health Australian National University Acton, Australian Capital Territory Australia; 3 Mental Health Policy, Brain and Mind Centre The University of Sydney Sydney, New South Wales Australia; 4 Thompson Institute University of the Sunshine Coast Moreton Bay, Queensland Australia; 5 Social Data Analytics Lab, Social Innovations Research Institute Swinburne University of Technology Hawthorne, Victoria Australia; 6 See Acknowledgements

**Keywords:** navigation, digital health, co-design, mental health care, health service planning, typology

## Abstract

**Background:**

Australia’s mental health care system has been characterized by complexity and fragmentation, as highlighted by numerous reports, commissions, and inquiries. In response, digital mental health care navigation tools have emerged as a promising solution to help individuals locate appropriate mental health services. The rapid proliferation of these tools—without a clear understanding of their definitions and characteristics—risks creating confusion rather than clarity for users. Terms such as “navigation” and “navigators” are often used interchangeably, further complicating the landscape.

**Objective:**

This study addressed the need for a standardized definition and typology of the characteristics of digital mental health care navigation tools.

**Methods:**

This study was part of the development of a digital mental health care navigation tool for navigators and planners (MChart). It used a co-design approach using expert-based cooperative analysis, which is a nominal group technique to develop a definition and typology of the characteristics of digital mental health care navigation tools. This process was guided by the Technology Readiness Level for Implementation Sciences framework. The co-design process involved two 2-hour sessions with an expert panel comprising 28 participants, including representatives from mental health planning, primary health care, health care financing and delivery, community-managed organizations, clinical settings (psychiatrists, psychologists, and general practitioners), and consumers.

**Results:**

The expert panel collaboratively developed a consensus definition of digital mental health care navigation tools, outlining their scope and intended targets. Through the co-design process, the panel identified 157 characteristics of digital mental health care navigation tools. These characteristics were organized into 5 primary domains: type, management, content, design, and quality. The definition and typology characteristics provide a structured framework for understanding and evaluating the diverse range of digital mental health care navigation tools currently available.

**Conclusions:**

The co-designed definition and typology offer a foundational step toward reducing confusion in the digital mental health care navigation space. This study supports the development of quality standards that can be used to assess and compare existing and future tools. This framework has the potential to guide developers, end users, and policymakers in creating more effective, user-centered navigation solutions within Australia’s mental health care system and internationally.

## Introduction

Australia’s mental health care system is fragmented and disorganized [1]. This is partly due to the split in responsibilities for mental health whereby the federal government manages primary mental health care services and states and territories fund hospital-based care. Community-based services are funded by federal, state, and territory governments, primary health networks (PHNs), and nonprofit organizations. Since the early 2020s, the proportion of funding allocated to mental health services within health funding has declined even as the COVID-19 pandemic significantly increased demand for these services in Australia and worldwide [2,3].

Australia’s efforts to improve its mental health care system have centered on the concept of stepped care, a model intended to clarify the type of care that people should receive and from whom [4]. Stepped care aims to match treatment intensity to clinical need while minimizing burden on the individual and the health system, thus optimizing effectiveness at the lowest appropriate cost and resource footprint. However, the stepped care model as applied in Australia has several limitations, including confusion between services and interventions and poor definitional clarity [5]. Unintended consequences of planning strategies, combined with increasing demand for and complexity of mental health care provision, make it harder for individuals to identify and access appropriate services. Subsequently, attention shifted to “integrated care,” a key focus of the National Mental Health and Suicide Prevention Agreement in 2022 [6], which is consistent with the World Health Organization global strategy on people-centered and integrated health services [7]. This approach aims to improve coordination across government, funders, services, and practitioners for a more cohesive and effective mental health care system. However, growth in demand and the complexity of mental health care provision is making it increasingly difficult for individuals to identify and access appropriate services. This can lead to confusion and frustration among help seekers and their families, as well as contributing to systemic inefficiencies such as inappropriate referrals and treatment delays. These inefficiencies may, in turn, contribute to downstream consequences, including productivity loss and increased costs.

In this context, improved service navigation has been recognized internationally as a priority [8,9]. Digital mental health care navigation tools are web-based resources designed to help individuals identify and access available services within their local areas. They have emerged as a key strategy to address the persistent fragmentation that characterizes Australia’s mental health care system. These tools can provide directories of services, referral pathways, geolocated service maps, diagnostic screening capabilities, and care pathways [10]. Examples in Australia include Healthengine, a national website targeting adults, consumers, youth, health professionals, and people with disabilities that helps in finding and booking an appointment with a health care provider.

Federal, state, and territory governments have committed to strengthening mental health care service navigation as part of broader efforts to enhance accessibility and integration. As part of this commitment, the Australian government allocated funding from 2023 to 2026 to modernize digital mental health care service system navigation and referral systems. This includes the development of a national framework for digital mental health care navigation [11,12]. In addition, the Productivity Commission’s inquiry into mental health, the National Suicide Prevention Adviser’s Final Advice, and the Royal Commission into Victoria’s Mental Health System have all recommended stronger service navigation systems [1,13,14]. National and local agencies, including PHNs and local health districts, have identified navigation as an unmet need [15,16].

The growing interest in digital mental health care navigation tools has generated new challenges. Tools have emerged rapidly and in an ad hoc fashion, developed in response to local needs but without national standards or consistent definitions. A recent infoveillance study using a systematic internet search to identify and describe digital mental health care navigation tools in Australia found that the number of these tools had increased from less than 10 to more than 100 within 4 years. Gaps and challenges identified included inconsistencies in inclusion criteria; a lack of comprehensive, regularly updated information, including estimated waiting times and availability of services to see new clients; limited accessibility and usability for diverse user groups; and the absence of integrated self-assessment or screening tools [10].

In addition, there is a notable lack of frameworks, policy, guidelines, and evidence-based recommendations to support the development, validation, and implementation of digital mental health care navigation tools. Currently, there is no common definition of what constitutes a digital mental health care navigation tool, limited clarity regarding the intended users for these tools, and little guidance on how to assess their quality. While the Australian Commission on Safety and Quality in Health Care provides voluntary standards for digital mental health services, these do not explicitly address digital mental health care navigation tools [17]. In addition, there is significant variability in how health care services and programs are described and delivered through mental health care navigation tools, contributing to a lack of definitional clarity and terminological inconsistency. For example, the term “navigation” (as a process) is often conflated with “navigators” (as agents delivering coordination services), as reflected in the broader literature on navigation models [18-22].

Establishing a shared language and consistent terminology is therefore a critical first step in the development of this emerging field. Such clarity can provide structure; reduce ambiguity; and support more coherent practice, policy, and research. An example of the relevance of classifications that has driven meaningful quality improvement is the development of the World Health Organization’s Family of International Classifications (WHO-FIC) [23]. The WHO-FIC brought consistency to the classification of diseases, defined the scope in the delivery of health programs, enabled global comparability of data, and supported evidence-based policy and planning. The establishment of the WHO-FIC led to the development of other international classifications for diseases (*International Statistical Classification of Diseases and Related Health Problems*); functioning, disability, and health (*International Classification of Functioning, Disability, and Health*); and health interventions (*International Classification of Health Interventions*) [24].

Developing a standardized definition of what constitutes a digital mental health care navigation tool, along with a clear description of its core components, can provide the foundation for future guidelines on the characteristics and quality standards of such tools. This paper addresses this gap by outlining a co-design process to develop a standardized definition and set of characteristics for digital mental health care navigation tools. The process used a nominal group technique to build consensus and guide decision-making, structured according to the subdomains of the Technology Readiness Level for Implementation Sciences (TRL-IS) framework [25-27]. The TRL-IS emphasizes the importance of involving both end users and experts throughout the development life cycle of health applications—including digital tools, interventions, and systems [28-30]. This approach extends beyond traditional co-design by promoting sustained engagement across all phases of innovation and implementation [27]. In the foundational stage of development (TRL-IS level 2), clarifying terminology and achieving consensus on core concepts is essential [28]. This step is particularly relevant in emerging areas such as digital mental health care navigation.

Establishing an agreed upon definition and typology of the characteristics of digital mental health care navigation tools enables more effective development, supports comparative evaluation, and underpins national standards for quality and effectiveness [31]. The goal is to inform planning, development, implementation, and quality assessment for future digital mental health care navigation tools in Australia and internationally, aligning with national quality and equity goals [1].

## Methods

### Overview

**Figure 1:**
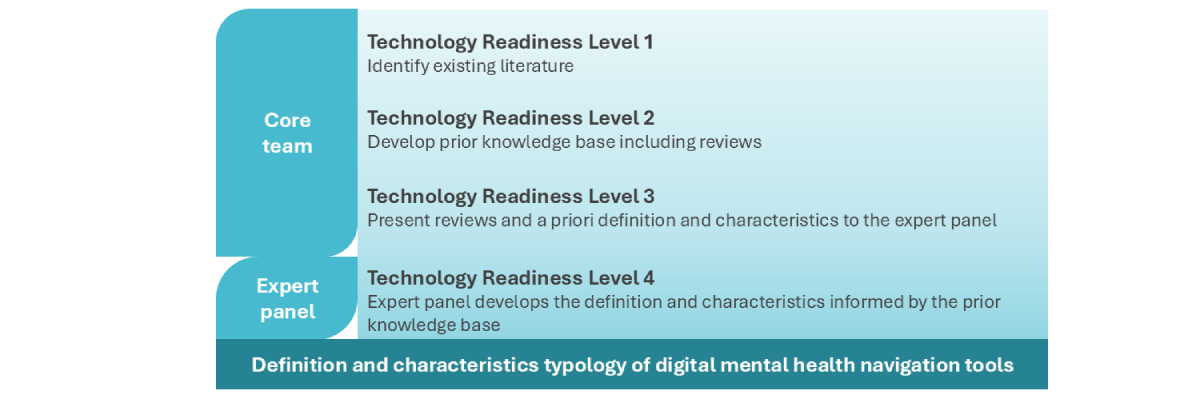
Development of the prior knowledge base for a co-design definition and typology of digital mental health care navigation tools using the Technology Readiness Level for Implementation Sciences framework.

The definition and typology were developed using a co-design approach as part of a broader project to create a digital mental health care navigation tool for health system planners (MChart) [32]. This approach used a nominal group technique guided by an expert-based cooperative analysis (EbCA) approach. EbCA is a structured participatory methodology that integrates existing evidence with stakeholder and expert knowledge to inform decision-making. Facilitated, iterative discussion conducted either face-to-face or virtually is used to support consensus on key decisions [33]. Development was guided by the TRL-IS, which has been adapted for use in health and social sciences contexts [25]. The TRL-IS incorporates multiple sources of knowledge, including scoping reviews and the combined expertise of the core project team and external subject matter experts. The process of establishing the prior knowledge base using TRL-IS levels involved identifying existing literature, developing reviews, presenting this to the expert panel, and developing the consensus definition and characteristics informed by the prior knowledge base. The process is outlined in Figure 1.

In this context, “expert knowledge” refers to a combination of professional and experiential insights, typically acquired through more than 10,000 hours of practice in a given field [34,35]. Contributions from individuals with lived experience ensures that the co-design process is meaningfully grounded in user-centered perspectives [34]. Expert knowledge was elicited through the nominal group technique, which is especially well suited to problem-solving in emerging fields in which knowledge is still developing, fragmented, or contested [36,37]. The method is widely used for the development of ontologies and typologies in such areas [38].

The core study team consisted of 7 multidisciplinary members, including clinicians (a psychiatrist, a mental health nurse, and an addiction specialist), health policymakers, and academic researchers. This team developed an a priori definition and set of characteristics for digital mental health care navigation tools drawing on recent reviews and studies [10,39]. To refine these preliminary outputs and build consensus, an expert panel was formed and engaged using a nominal group technique.

Participants were invited based on their professional affiliations and relevant expertise. All panel members had experience working within the mental health care system and knowledge of the challenges associated with digital navigation and service access. Invitations were sent via email, and those who accepted were included in the panel. In total, 31 individuals were purposively recruited, with 28 (90.3%) accepting the invitation to participate. The panel included a mental health care consumer and advocate; mental health planning and policy practitioners; and representatives from PHNs, mental health community-managed organizations, health care financing and delivery, and clinicians (including psychiatrists, psychologists, general practitioners, and a nurse health care system navigator). The full list of panel members and their demographics and expertise are provided in Multimedia Appendix 1. The expert panel was chaired by the chief investigator (a psychiatrist and researcher). The panel convenor was a researcher with mental health policy expertise.

Between November 2023 and December 2023, the expert panel convened twice for 2-hour meetings conducted in hybrid format (in person and online via Zoom [Zoom Video Communications]). Consistent with the EbCA approach, 1 week prior to the meetings, background documents and questions were circulated to members to enable preparation and support informed participation. All meetings were recorded and transcribed, with notes and key decisions circulated to panel members for validation and feedback. Each session began with an overview of the expert panel’s purpose followed by a presentation from the core study team of their a priori definition (Multimedia Appendix 2) and characteristics (Multimedia Appendix 3).

The following questions were posed to the expert panel: (1) “Do you agree with the proposed definition of a digital mental health care navigation tool?” (2) “Do you agree with the identified characteristics and domains of digital mental health care navigation tools?” (3) “Do you agree with the proposed characteristics, domains and subdomains of digital mental health care navigation tools?”

In line with the EbCA approach, the nominal groups used a structured, iterative method for consensus building. Participants were provided with a synthesis of relevant evidence and a set of guiding questions to support informed, individual reflection. During facilitated discussion, participants shared, discussed, and refined their perspectives through collective deliberation, integrating the evidence with experiential knowledge. Discussions were recorded and synthesized into meeting notes by the study team, with iterative updates to the definition, domains, and characteristics circulated to participants between meetings. Particular attention was paid to the contributions from panel members representing consumer and carer organizations, ensuring that their perspectives were meaningfully incorporated into the consensus process.

On the basis of panel input, the core study team revised the definition and characteristics, which were then circulated for further feedback to refine the consensus definition and typology. Panel members provided feedback either during meetings or individually via email if they preferred. Discussions focused on mental health care provision for individuals with mental health conditions. Topics related to general public information provision, prevention, and mental health interventions were excluded from the scope.

### Ethical Considerations

The study protocol was reviewed and approved by the Australian Capital Territory (ACT) Health Human Research Ethics Committee (2023.ETH.00112). Participants were provided with an information sheet and were asked to complete and sign a consent form and a conflict-of-interest declaration prior to their participation in the expert panels. Consistent with the nominal group technique, a conflict of interest did not preclude inclusion in the expert panels. Declared conflicts included employment in organizations involved in mental health service planning in the ACT (2/28, 7.1%) and employment in an organization that contributed funding for the project (1/28, 3.6%). All data reported in public outputs, including reports and publications, were deidentified. Panel members were not compensated for their time, with the exception of the lived experience consumer representative, who received an honorarium for their contribution.

The study protocol was not prospectively registered. The methodology was guided by the ACCORD (Accurate Consensus Reporting Document) reporting template for consensus-based methods, and the checklist is provided in Multimedia Appendix 4 [40].

## Results

Expert panel meetings were conducted in October 2023 and November 2023. Three hours and 50 minutes of expert panel meetings were recorded, transcribed, and summarized into dot points to support iterative feedback and validation by the panel members.

### Definition

The study team presented their draft definition of digital mental health care navigation tools to the expert panel, who were invited to respond to the guiding questions outlined earlier. This discussion generated several key considerations, including clarification of the term “individuals,” the relationship between digital navigation tools and mental health interventions, and the intended purpose of these tools. Panel members emphasized the importance of using nonmedicalized language and debated whether health promotion and prevention should be included as components of mental health care. Following these discussions, the study team revised the definition in collaboration with the panel. The co-designed definition, shown in Textbox 1, identifies the primary function of digital mental health care navigation tools as supporting various users in locating appropriate mental health care services and professionals. The key characteristic of a digital mental health care navigation system is that it provides accessible, service-specific information to facilitate digital mental health care navigation.

**Definition of digital mental health care navigation tools.**

**What are digital mental health care navigation tools?**

- Digital mental health care navigation tools are online technologies and software applications that help find and access available mental health care services and professionals.
- Digital mental health care navigation tools can be a stand-alone online system or can comprise modules or other online tools for information provision, screening, diagnosis, and treatment.
- These tools can be used by help seekers, health care professionals and support workers, care coordinators, and navigators to provide recommendations, guide referrals, and develop care pathways.
- Planners and managers can use digital mental health care navigation tools to facilitate planning, needs assessment, and decision-making.
- A navigation tool is not (1) a navigator, (2) a care intervention (eg, counseling), (3) a diagnosis and assessment tool, or (4) a service.

### Characteristics

#### Overview

The study team presented an initial set of proposed characteristics for digital mental health care navigation tools to the expert panel based on a hierarchical tree. This generated discussion on the conceptual frameworks underpinning the typology, the intended users of digital mental health care navigation tools, and their core purpose and goals—including potential benefits and ethical considerations. The panel also had an in-depth discussion on the terminology used to describe individuals, carers, and professionals, emphasizing the importance of inclusive and respectful language.

The draft typology presented to the expert panel included 5 broad characteristic domains: type, service information, design, quality, and implementation and management, encompassing 77 domains and subdomains. On the basis of panel feedback, the “implementation” domain was integrated into the “quality” domain, “service information” was changed to “content,” and the number of domains and subdomains was expanded to 157.

The final set of characteristic domains and subdomains developed through this consensus process is shown in Textbox 2. The full set of characteristic domains, subdomains, sub-subdomains, and fourth- and fifth-level domains can be found in Multimedia Appendix 5.

The final typology of digital mental health care navigation tools includes 5 broad characteristic domains: type, management, content, design, and quality. These 5 domains encompass 27 subdomains, 72 sub-subdomains, 45 fourth-level domains, and 13 fifth-level domains as identified through expert panel discussions.

**Characteristics of digital mental health care navigation tools.**

**Type**

- Purpose and activity
- Target
- Scope
- Functions

**Management**

- Authority
- Funding source
- Area of coverage
- Organization
- Moderation
- Data management
- Advertising

**Content**

- Basic information
- Access
- Waiting list
- Health care provider and organization
- Types and process of care provision
- Cost or fee

**Design**

- Development
- Technical aspects
- Data sources

**Quality**

- Glossary and definitions used
- Classifications used
- Implementation
- Information quality
- Credibility
- Quality standards
- Currency and updates
- Safety, security, privacy and respect

#### Type

The “type” domain included 4 subdomains, 15 sub-subdomains, and 6 fourth-level domains covering the tool’s purpose, target users, scope, and functions. Extensive discussion focused on the terminology used to describe target populations. In particular, the term “carer” was identified as ambiguous as it can refer both to unpaid family members supporting individuals with mental health conditions and to paid professionals. Reaching consensus on inclusive, precise language for different user groups was a key challenge for the panel.

#### Management

The “management” domain included 7 subdomains and 2 sub-subdomains, which covered the governance and operational oversight of digital mental health care navigation tools. Aspects of management included the identification of the funding sources, area of geographic coverage, organizational structure, content moderation processes, data management protocols, and approaches to advertising. The panel underscored the need for transparent and accountable management practices to ensure the tool’s integrity and sustainability.

#### Content

“Content” included 5 subdomains, 22 sub-subdomains, 7 fourth-level domains, and 2 fifth-level domains, which referred to the nature and comprehensiveness of the information provided about available services. Key elements included service access, health care organization, types of care, access, and cost. The panel emphasized that comprehensive and reliable service information is central to a digital mental health care navigation tool’s usefulness. The quality of the service directory component, particularly the inclusion of up-to-date and detailed information, was identified as essential.

#### Design

The “design” domain included 3 subdomains, 11 sub-subdomains, 18 fourth-level domains, and 9 fifth-level domains that encompassed the development process, technical aspects, and data sources underlying the digital mental health care navigation tool. Technical aspects included the software application, interactivity, search capabilities, interoperability, and user aids. Accessibility features such as multilingual support, plain-language options, and screen readers were highlighted as necessary for ensuring that the tool can serve a wide range of users. Panel members also stressed the importance of transparency about data sources.

#### Quality

The “quality” domain incorporated 8 subdomains, 22 sub-subdomains, 14 fourth-level domains, and 2 fifth-level domains that included the use of glossaries and definitions; information quality; credibility; adherence to quality standards; currency of updates; and considerations of safety, security, privacy, and respect. The expert panel placed emphasis on the importance of quality assurance mechanisms, particularly the accuracy and currency of the information provided. Ethical considerations including user privacy, data security, and respectful representation were highlighted as key to maintaining user trust and safety.

## Discussion

### Principal Findings

To our knowledge, this is the first definition and typology of digital mental health care navigation tools. Despite the growing number of digital mental health care navigation tools in Australia and internationally, there remains a limited body of peer-reviewed literature addressing their formal definition, structure, and quality. This study contributes to this rapidly emerging and important field by developing a co-designed definition and typology of characteristics for digital mental health care navigation tools.

The comprehensive and structured approach used in this study is well suited to the task of developing foundational knowledge. The use of the nominal group technique proved to be a viable and acceptable method for achieving consensus. This is consistent with its application in other health-related domains, including brain injury case management, clinical interventions, education and training, practice development, clinical measures, and health care research [41-43].

A key contribution of this study is the development of a shared language for digital mental health care navigation tools. Establishing a common vocabulary can help reduce ambiguity (where terms are interpreted in different ways) and vagueness (where definitions lack specificity) [44]. This is particularly important due to inconsistency in currently used terminology. For example, individuals interacting with mental health care services may be described as “clients,” “customers,” “consumers,” “users,” “patients,” or “carers.” Similarly, workforce personnel may be referred to as “support workers,” “recovery workers,” or “case workers”—raising questions about whether these roles provide similar types of support. Comparable issues arise in the way in which services and systems are described, reflecting broader challenges with terminology in health systems research [45,46]. Without greater conceptual clarity, it is difficult to assess and compare the quality, purpose, or functionality of digital mental health care tools.

Lack of consensus in naming has far-reaching implications across clinical, administrative, research, and policy contexts, including information provision, screening, or clinical interventions [45]. Clinicians and help seekers may use and interpret names differently. Lack of agreement can result in inconsistent service delivery as terms may be applied differently, leading to variability in access. These challenges highlight the need to develop a glossary of terms to clarify and explain the components of digital mental health care navigation tools.

The typology developed in this study lays the groundwork for the quality assessment of digital mental health care navigation tools. However, the absence of standardized definitions and quality criteria currently limits the ability to evaluate their reliability, accuracy, and ethical design. While the National Safety and Quality Digital Mental Health Standards [17] provide voluntary guidance for digital mental health services, they do not explicitly include criteria for digital navigation tools. The standards recommend that service providers implement quality improvement systems, but they do not define what constitutes quality or accuracy in the context of information provision [10]. The need for tailored quality assessment mechanisms to digital mental health care navigation tools was emphasized by the expert panel.

### Strengths and Limitations

This study is the first to develop a co-designed definition of digital mental health care navigation tools and identify their characteristics through a structured expert consensus process. By engaging a diverse panel of stakeholders with expertise in mental health policy, service delivery, research, clinical practice, and lived experience, this study strengthens the relevance and applicability of its findings across contexts [35].

The involvement of expert knowledge in defining and characterizing complex constructs has been shown to enhance the rigor and utility of scientific knowledge development [35]. The resulting definition and typology have the potential to inform the development, evaluation, planning, funding, and quality assessment of digital mental health care navigation tools and digital navigation tools more generally. For policymakers and planners, these outputs offer the foundation for a more precise categorization of tools, clearer understanding of service gaps and overlaps, and use of more consistent terminology within the digital mental health care ecosystem.

Panel members’ individual perspectives, experiences, or professional affiliations may have influenced group deliberations. However, this was balanced with the careful and purposeful consideration of the composition of the panel to include diverse perspectives and expertise, including representation from government, clinical care, community services, and lived experience. Furthermore, consistent with nominal group protocols, the group discussion was facilitated such that all opinions were heard, and there was a process of openly reaching a consensus through rounds of discussion within the group. Conflict-of-interest declarations were also collected.

While the nominal group–informed expert panel provides valuable insights from experts, it is important to acknowledge that the perspectives gathered may not be fully representative of the broader community of mental health care policymakers and practitioners. Transparent reporting of the panel’s compositing and expertise improves reproducibility and credibility [47]. The panel included a high percentage of panelists from the ACT as this was the pilot area and local knowledge is critical for the tool’s assessment and validation. Experts from 4 states and territories participated, and as the ACT is the capital of Australia and the location of headquarters of governmental agencies, many participants had previous or current experience working in other states as well as in national organizations. The expert panel included 1 consumer representative with unique experience in digital health, having been a deputy chair of the National Safety and Quality Digital Mental Health Standards committee. In addition, 2 expert panel members represented national organizations and peak bodies of mental health care providers and consumers.

Future work is needed to evaluate the generalizability, usability, and adoption of the proposed definition and typology characteristics of digital mental health care navigation tools in diverse contexts. Our previous development of taxonomies for mental health services (Description and Evaluation of Services and Directories [48], a brain injury case management taxonomy [42], and the Global Impact Analytics Framework [27]) illustrate how taxonomies can be generalized to other fields. The Description and Evaluation of Services and Directories has been extended to drug and alcohol, aging, disability, and Indigenous health and neurology and has been used in 35 countries. The brain injury case management taxonomy has been used in disability and chronic care [42], and the Global Impact Analytics Framework has been used in impact analysis of digital mental health interventions for veterans and first responders [49] and for First Nations social and emotional well-being [50]. It would also be important to run an analysis of digital mental health care navigation tools using this definition to delineate between digital navigation tools and those that are not navigation tools, such as those limited to information provision, clinical assessment, or interventions. To improve generalizability, in the future, it would be important to develop a related glossary of terms to further support consistent use.

The consensus on digital mental health care navigation tool characteristics is critical for the development of quality indicators to allow for systematic assessment and benchmarking. While the definition and typology developed in this study have potential applicability beyond Australia and across health systems more broadly, it is important to consider the extent to which specific elements may be shaped by the Australian policy and service context. For example, features related to system navigation, service categorization, and referral pathways are influenced by the structure of Australia’s mixed public-private health care system, the role of federally funded digital mental health services, and the presence of national initiatives such as Medicare Mental Health. In health systems with different funding arrangements, levels of service integration, or digital infrastructure, these elements may require adaptation. At the same time, several core components of the typology—such as the distinction among navigation, information provision, assessment, and intervention functions; the emphasis on user-centered design; and the role of navigation tools in addressing fragmentation—are likely to be transferrable across contexts. These features reflect common challenges in mental health systems internationally, including complexity, fragmentation, and barriers to access [51,52].

Given that digital mental health tools are implemented across diverse systems with varying infrastructure and governance arrangements [53,54], future research should focus on assessing external validity and applicability across settings, including through cross-country consensus approaches [38,55]. Such work would help distinguish context-specific versus universal elements and support the development of a globally relevant framework for digital mental health care navigation tools.

### Conclusions

Digital mental health care navigation tools play a significant role in addressing persistent challenges in Australia’s mental health system, particularly fragmentation, delayed access to care, and unmet needs. The rapid proliferation of digital mental health care navigation tools reflects a growing momentum across sectors to invest in digital infrastructure that helps people connect with services more efficiently. However, the rapid rollout of these tools in the absence of shared definitions, standards, or quality frameworks risks further entrenching the very fragmentation they are intended to resolve. Without clear boundaries regarding purpose, audience, and functionality, digital mental health care navigation tools may become inconsistent in design and messaging, potentially leading to confusion for users and reduced effectiveness overall. Over time, such inconsistency may undermine trust and diminish the perceived value of digital mental health care navigation tools as important infrastructure for mental health reform.

This study directly addresses that risk by offering a co-designed definition and typology of characteristics of digital mental health care navigation tools. These foundational elements provide a starting point for greater coherence, comparability, and accountability in the development and evaluation of such tools. They also enable a more consistent language for policy and planning, which is essential for system-level coordination.

For policymakers, health system managers, and funders, these findings underscore the need to establish quality standards and oversight mechanisms specific to digital mental health care navigation tools. These standards should include guidance on terminology, data integrity, ethical design, accessibility, and transparency of service information. Without such measures, digital tools risk becoming fragmented solutions within a fractured system. Ultimately, effective digital mental health care navigation tools must do more than provide information; they must support individuals, families, carers and kin, and communities in making sense of a complex and often overwhelming system. When designed with clarity, care, and accountability, digital mental health care navigation tools can play a transformative role in improving access, equity, and mental health care outcomes across Australia and internationally.

## Appendix

Multimedia Appendix 1 Expert panel demographics and expertise.

Multimedia Appendix 2 A priori definition.

Multimedia Appendix 3 A priori characteristics.

Multimedia Appendix 4 ACCORD checklist.

Multimedia Appendix 5 Full set of digital mental health care navigation tool characteristic domains and subdomains.

## Abbreviations

ACCORD: Accurate Consensus Reporting Document
ACT: Australian Capital Territory
EbCA: expert-based cooperative analysis
PHN: primary health network
TRL-IS: Technology Readiness Level for Implementation Sciences
WHO-FIC: World Health Organization’s Family of International Classifications

## Data Availability

The data are not publicly available due to privacy and ethical restrictions but may be obtained from the corresponding author on reasonable request and with permission from the institutional review board.
