# Supplementary material for: Structuring Digital Mental Health Care Navigation: Co-Design Nominal Group Technique Study to Develop the MChart Definition and Typology of the Characteristics of Digital Mental Health Care Navigation Tools
Source: JMIR Ment Health. 2026 Jul 27;13:e91462. doi: 10.2196/91462 (PMC13458349; doi:10.2196/91462)
Supplement: Supplementary file 2 [file mental_v13i1e91462_app1.docx]

Supplementary File 1: Expert panel members demographics and expertise

| **Gender** | **Role/Expertise (at the time the Panels were conducted)** | **State** |
| --- | --- | --- |
| Female | Health Services Planning | ACT |
| Male | Primary health network practitioner | ACT |
| Female | Primary health network practitioner | Victoria |
| Female | Health service planning | ACT |
| Female | Mental health policy practitioner | ACT |
| Male | Mental health community managed organisation representative | ACT |
| Male | Psychologist | ACT |
| Female | Psychologist | ACT |
| Male | Mental health policy practitioner, Registered Nurse, business analysist | ACT |
| Female | Lived experience consultant | NSW |
| Female | Psychiatrist, mental health policy practitioner | ACT |
| Male | Mental health community managed organisation representative | ACT |
| Female | General Practitioner | ACT |
| Female | Primary health network practitioner | ACT |
| Female | Mental health community managed organisation representative | ACT |
| Female | Psychiatrist/Mental health policy practitioner | ACT |
| Female | Psychologist | ACT |
| Male | Health service planning | ACT |
| Male | Primary health network practitioner | Western Australia |
| Female | Psychiatrist | ACT |
| Male | Health care financing and delivery | Victoria |
| Male | Mental health community managed organisation | ACT |
| Male | Mental health policy practitioner | ACT |
| Male | Primary health network | ACT |
| Female | Health care financing and delivery | Victoria |
| Female | General Practitioner | ACT |
| Female | Mental health community managed organisation | ACT |
| Female | Nurse, health care system navigator | ACT |
