# Supplementary material for: Structuring Digital Mental Health Care Navigation: Co-Design Nominal Group Technique Study to Develop the MChart Definition and Typology of the Characteristics of Digital Mental Health Care Navigation Tools
Source: JMIR Ment Health. 2026 Jul 27;13:e91462. doi: 10.2196/91462 (PMC13458349; doi:10.2196/91462)
Supplement: Supplementary file 3 [file mental_v13i1e91462_app2.docx]

A Priori Definition

*Mental health navigation tools refer to resources, tools, and technologies that help individuals navigate the complex and often confusing landscape of mental health services and resources. These tools can include online directories of mental health services, mobile apps for tracking mental health symptoms, chatbots and virtual assistants for providing support and guidance, and peer support networks.*
